# Supplementary material for: Magnetically propagating Hund’s exciton in van der Waals antiferromagnet NiPS3
Source: Nat Commun. 2024 Apr 25;15:3496. doi: 10.1038/s41467-024-47852-x (PMC11045826; doi:10.1038/s41467-024-47852-x)
Supplement: Supplementary file 1 — Supplementary information [file 41467_2024_47852_MOESM1_ESM.pdf]

Supplementary Information for: Magnetically Propagating Hund's exciton in van der Waals antiferromagnet $NiPS_3$

W. He,* Y. Shen, K. Wohlfeld, J. Sears, J. Li, J. Pelliciari, M. Walicki, S. Johnston, E. Baldini, V. Bisogni, M. Mitrano, and M. P. M. Dean†

Contents

Supplementary Note 1. Exact diagonalization calculations 1
A. Cluster model 1
B. Determining the cluster model parameters 2
C. Anderson impurity model 3
D. Single site atomic model 3
E. Discussion of an alternative model 4
F. Comparison of all the models 5

Supplementary Note 2. Wavefuction analysis 5

Supplementary Note 3. Detailed fitting procedures for the resonant inelastic x-ray scattering (RIXS) spectra 6
A. Energy zero determination 6
B. Best fits to the low temperature momentum-dependent RIXS spectra 7
C. Cross-checks of the exciton and double-magnon dispersions 7
D. Magnon and double-magnon fits with Voigt functions 7
E. Fits to the high-temperature spectra 8
F. Fits to the incident energy dependent spectra 8

Supplementary Note 4. Double-magnon and exciton propagation 8
A. Double-magnon propagation 8
B. Exciton hopping 9
C. Exciton propagation 9

Supplementary Note 5. Tight-binding model fit to the exciton dispersion 10

References 24

Supplementary Note 1. Exact diagonalization calculations

Here we outline the detailed approach and values used in our RIXS calculations. Throughout this section, we represent the model in hole (rather than electron) language.

A. Cluster model

Our cluster representation of $NiPS_3$ uses six S atoms surrounding a central Ni atom. The orbital basis includes the full set of ten Ni $3d$ spin-orbitals and six S $3p$ spin-orbitals for each of the six neighboring S ions. The Ni $2p_{3/2}$ core

* whe1@bnl.gov
† mdean@bnl.gov

Supplementary Table I. **Full list of parameters used for the NiS_6 cluster model in the ED calculations.** Units are eV.

On-site orbital energies and spin-orbit coupling						On-site Coulomb interactions				
$\epsilon_d(e_g)$	$\epsilon_d(t_{2g})$	ϵ_p	ζ_i	ζ_n	ζ_c	F^0_{dd}	F^2_{dd}	F^4_{dd}	F^0_{pp}	F^2_{pp}
0	0.42	7.5	0.083	0.102	11.4	7.88	10.68	6.68	3.2	5
Hopping integrals		Intersite Coulomb interactions				Core-hole potential				
$V_{pd\sigma}$	$V_{pp\sigma}$	F^0_{dp}	F^2_{dp}	G^1_{dp}	G^3_{dp}	F^0_{dp}	F^2_{dp}	G^1_{dp}	G^3_{dp}	
0.95	0.8	1	0	0	0	7.45	6.56	4.92	2.80	

states are included to simulate the resonant pathway involved in RIXS. The Hamiltonian for this model is

$$\mathcal{H} = \hat{E}_d + \hat{E}_p + \hat{U}_{dd} + \hat{U}_{pp} + \hat{U}_{dp} + \hat{U}_q + \hat{T}_{pp} + \hat{T}_{dp} + \hat{\zeta}, \tag{1}$$

which includes the on-site energies $\hat{E}_d$ and $\hat{E}_p$ for the Ni $3d$ and S $3p$ orbitals, respectively. Here, $\hat{U}_{dd}$ and $\hat{U}_{pp}$ are on-site Coulomb interactions and $\hat{U}_{dp}$ are the intersite interactions, and $\hat{U}_q$ is the interaction between the core and valence holes. Regarding the intersite hybridization, $\hat{T}_{pp}$ indicates p–p hopping among S sites and $\hat{T}_{dp}$ stands for p–d hopping between Ni and S sites. The charge-transfer energy Δ is included implicitly as discussed below. $\hat{\zeta}$ represents spin-orbit coupling, which we include for the core and valence states.

The crystal field associated with the trigonal distortion in $NiPS_3$ is on the order of only 1 meV [1, 2], We therefore assumed cubic symmetry for simplicity. The $t_{2g} - e_g$-orbital energy splitting is further set by the Ni crystal field, and is specified by $10D_q$. To describe Ni-S hopping, we use the Slater-Koster parameters of $V_{pp\sigma}$, $V_{pp\pi}$, $V_{pd\pi}$, and $V_{pd\sigma}$, which denote hopping between Ni d and S p states with either π or σ orbital symmetry. We denote the energy of the S $3p$ orbitals measured relative to the Ni e_g orbitals by ϵ_p and neglect the splitting between the $3p_\sigma$ and $3p_\pi$ orbitals in the cluster.

The Coulomb interactions in the model are specified using Slater integrals. Following standard methods, we include F^0_{dd}, F^2_{dd}, and F^4_{dd} to describe interactions on Ni $3d$ orbitals. Similarly, we used F^0_{pp} and F^2_{pp} for S $3p$ orbitals. F^0_{dp} is the intersite Coulomb interaction between Ni $3d$ and S $3p$ orbitals. In the RIXS intermediate states, we used F^0_{dp}, F^2_{dp}, G^1_{dp}, and G^3_{dp} to describe the Coulomb interactions between Ni $3d$ and Ni $2p$ holes. Lastly, we also included spin-orbit coupling terms of Ni $3d$ orbitals for the initial and intermediate states (ζ_i and ζ_n), as well as the much larger core-hole coupling (ζ_c). An inverse core-hole lifetime $\Gamma_c = 0.6$ eV half-width at half-maximum (HWHM) was used in order to fit the observed width of the resonance and the final state energy loss spectra are broadened using a Gaussian function with a full-width at half-maximum (FWHM) of 0.05 eV, in order to match the observed width of the final states.

B. Determining the cluster model parameters

Although there are several parameters in the original Hamiltonian, they can be constrained by physical considerations and by exploiting the richness of the $NiPS_3$ RIXS spectra. We start by outlining the different constraints on the parameters:

1. **Coulomb interactions**: The Slater integrals for Ni $3d$ Coulomb interactions can be recast into onsite Coulomb repulsion U and Hund's coupling J_H. Since we work in the hole language, the on-site Coulomb repulsion for the ligands is not crucial since double hole occupation on ligands is unlikely. Indeed, we get nearly zero probability for the $d^{10}\underline{L}^2$ ($\underline{L}$ stands for a ligand hole) configuration in our calculations, as shown in Fig. 1e in the main text. Therefore, F^0_{pp} and F^2_{pp} do not influence our conclusions and are fixed to standard values appearing in the literature [3]. Due to the distance between the Ni and S atoms the strength of the intersite Coulomb interaction F^0_{dp} is expected to be much smaller than the Ni on-site Coulomb interactions and thus plays a negligible role. We therefore fix this value to 1 eV, consistent with values often found in the literature [4]. The precise value of this interaction is not crucial because its effect on the charge-transfer energy can also be absorbed into the S $3p$ orbital energy ϵ_p.

Supplementary Table II. **Projection of the NiS_6 cluster model to the AIM.** Here, ϵ_L refers to the ligand orbital energies determined by the S $3p$ orbital energy ϵ_p and $T_{pp} = |V_{pp\sigma} - V_{pp\pi}|$. The hopping integrals V between Ni $3d$ orbitals and ligand orbitals can be evaluated from p–d hoppings. Units are eV.

Symmetry	ϵ_d	ϵ_L	V
e_g	$\epsilon_d(e_g)$	$\epsilon_p - T_{pp}$	$\sqrt{3}\|V_{pd\sigma}\|$
t_{2g}	$\epsilon_d(t_{2g})$	$\epsilon_p + T_{pp}$	$2\|V_{pd\pi}\|$

2. **Hopping integrals**: Making use of the Slater-Koster scheme, all the hopping integrals can be derived from two parameters, $V_{pd\sigma}$ and $V_{pp\sigma}$. Here, we fixed $V_{pd\pi} = -V_{pd\sigma}/2$ and $V_{pp\pi} = -V_{pp\sigma}/4$, which is common for transition metal compounds [5].

3. **Charge-transfer energy**: In a multi-orbital system, this energy is defined as $\Delta = \epsilon(d^9\underline{L}) - \epsilon(d^8) = [\epsilon_d(e_g) + \epsilon_p + F^0_{dp}] - [\epsilon_d(e_g) \times 2 + F^0_{dd} - 8F^2_{dd}/49 - F^4_{dd}/49]$. Once the Ni $3d$ on-site Coulomb interactions and $10D_q$ are chosen, the charge-transfer energy is only affected by the energy difference between the S $2p$ states and the Ni $3d$ e_g states, i.e. ϵ_p (recall we set the latter to zero).

4. **Core-hole potential**: The core-hole interactions are known to be only weakly screened in the solid state. We initially set the values to 80% of their atomic values and further refined them based on the x-ray absorption spectrum (XAS) (Supplementary Fig. 1) after we determined other above parameters from the RIXS spectra.

5. **Spin-orbit coupling**: The spin-orbit coupling terms for the Ni $3d$ orbitals are weak and have negligible effects of the spectra. We consequently simply fixed them to their atomic values. The core-hole spin-orbit coupling parameter is adjusted slightly to match the L_2 peak position.

Aside from the core-hole potential, we have $10D_q$, U, J_H, $V_{pd\sigma}$, $V_{pp\sigma}$, and ϵ_p as the only free parameters, which have distinct effects on the RIXS spectra. The energy of the excitation around 1.0 eV is mostly sensitive to J_H, while the position of the feature around 1.1 eV is primarily determined by $10D_q$. The exciton energy is correlated with a combination of J_H, $10D_q$, and U. Once J_H and $10D_q$ are determined by the excitations around 1.0 and 1.1 eV, respectively, we can tune the onsite Coulomb repulsion U to match the exciton energy. ϵ_p is constrained by the resonant energy as well as the intensity of the exciton, while the hopping integrals can be determined mainly by the resonant energy of satellite peaks. Using the above strategy, we successfully determine these parameters with estimated error bars of ~ 1 eV for all the Coulomb interactions and ~ 0.2 eV for hopping integrals and $10D_q$. The final parameters are provided in Tab. I.

C. Anderson impurity model

The NiS_6 cluster model can be effectively projected to an Anderson impurity model (AIM) using ligand field theory [6, 7]. The S $3p$ orbitals form 10 ligand orbitals that have the same symmetry as the Ni $3d$ orbitals through p–p hopping. The ligand orbitals further hybridize with the Ni $3d$ orbitals, which can be parameterized based on p–d hopping integrals. Correspondingly, the energies of the ligand orbitals and their hopping integrals with the Ni $3d$ orbitals can be evaluated as shown in Tab. II. The missing piece is the onsite Coulomb interactions for the ligand orbitals, and we refined these interactions using the same approach as for the cluster model. The full list of the parameters are shown in Tab. III. We will show later that the AIM calculation results are, as expected, essentially identical to the NiS_6 cluster results.

D. Single site atomic model

To test whether a simpler model might be able to capture the relevant physics, we also considered an atomic model composed of a single Ni atom with 10 effective Ni $3d$ orbitals. In this case, these spin orbitals represent effective hybridized Ni-S orbitals, so they are consequently quite different from the Ni orbitals in the above models. As expected, with appropriate values of crystal field and Hund's coupling, this model has a triplet ground state and a singlet excited

Supplementary Table III. **Full list of parameters used for the AIM in the ED calculations.** Here, ζ_{L} is the spin-orbit coupling strength for the ligand orbitals, U_{dL} is intersite Coulomb interactions, and F^0_{LL}, F^2_{LL}, and F^4_{LL} are the Slater integrals for ligand orbital onsite Coulomb interactions. Units are eV.

On-site orbital energies					Spin-orbit coupling						Hopping integral
$\epsilon_d(e_{\mathrm{g}})$	$\epsilon_d(t_{2\mathrm{g}})$	$\epsilon_L(e_{\mathrm{g}})$	$\epsilon_L(t_{2\mathrm{g}})$		ζ_{i}	ζ_{n}	ζ_{c}	ζ_{L}			$V_{pd\sigma}$
0	0.42	6.5	8.5		0.083	0.102	11.4	0			0.95
Coulomb interactions								Core-hole potential			
F^0_{dd}	F^2_{dd}	F^4_{dd}	F^0_{LL}	F^2_{LL}	F^4_{LL}	U_{dL}		F^0_{dp}	F^2_{dp}	G^1_{dp}	G^3_{dp}
7.88	10.68	6.68	0.46	2.59	1.62	1		7.45	6.56	4.92	2.80

Supplementary Table IV. **Full list of parameters used for the single site atomic model in the ED calculations.** All parameters are in units of eV.

$\epsilon_d(e_{\mathrm{g}})$	$\epsilon_d(t_{2\mathrm{g}})$	ζ_{i}	ζ_{n}	ζ_{c}	F^0_{dd}	F^2_{dd}	F^4_{dd}	F^0_{dp}	F^2_{dp}	G^1_{dp}	G^3_{dp}
0	1.07	0.083	0.102	11.2	-0.70	5.26	3.29	0.26	6.18	2.89	1.65

state. We refined the parameters using a methodology similar to the one we used for our cluster model. However, we found that the exciton energy could not be accurately reproduced with this model, which we will discuss in more depth when we compare the models. The obtained values of these parameters are listed in Tab. IV.

E. Discussion of an alternative model

Reference [8] (which we will refer to as Ref. "A") used a NiS_6 cluster to calculate the RIXS spectra as we did here but adopting different parameters. Most of the parameters are listed in that paper but two of them are not, namely the energy different between Ni e_{g} and S $3p$ orbitals and the core-hole potential parameter F^0_{dp}, which can be indirectly inferred from $\Delta = \epsilon(d^9\underline{L}) - \epsilon(d^8) = 0.95$ eV and $\Delta' = \epsilon(d^{10}\underline{cL}) - \epsilon(d^9\underline{c}) = -0.55$ eV where $\underline{c}$ stands for a core hole. It can be easily concluded that $\epsilon_p - \epsilon_d(e_{\mathrm{g}}) = 6.37$ eV but it is tricky to obtain F^0_{dp} since it depends on how core-hole potential is included in Δ'. We choose it to be 7.79 eV. Similarly, we inferred that a similar (but not identical) inverse core-hole lifetime of 0.4 eV HWHM was used and their spectra were broadened using a Lorentzian function with FWHM of 0.1 eV. Table V lists all the parameters. We have confirmed that our approach reproduces Ref. A's results [8] when we adopt their parameters.

Supplementary Table V. **Full list of parameters used for the NiS_6 cluster model in Ref. A [8].** The italic values are parameters that are not directly listed in the paper but inferred from other parameters. Units are eV.

On-site orbital energies and spin-orbit coupling							On-site Coulomb interactions				
$\epsilon_d(e_{\mathrm{g}})$	$\epsilon_d(t_{2\mathrm{g}})$	ϵ_p	ζ_{i}	ζ_{n}	ζ_{c}		F^0_{dd}	F^2_{dd}	F^4_{dd}	F^0_{pp}	F^2_{pp}
0	1	*6.37*	0.08	0.08	11.5		8	14.68	9.12	1	0
Hopping integrals			Intersite Coulomb interactions					Core-hole potential			
$V_{pd\sigma}$	$V_{pd\pi}$		F^0_{dp}	F^2_{dp}	G^1_{dp}	G^3_{dp}		F^0_{dp}	F^2_{dp}	G^1_{dp}	G^3_{dp}
0.74	-0.4		0	0	0	0		*7.79*	6.18	4.63	2.63

F. Comparison of all the models

Supplementary Fig. 2 summarizes the calculation results for all the three models, as well as the one from Ref. A [8]. The exciton at around 1.47 eV can only be captured in the models with ligand orbitals. Compared with the combination of parameters used in Ref. A [8], our model (NiS_6 cluster or equivalently AIM) better captures the *dd* excitations at 1.0 and 1.1 eV. We also see that our model has the secondary satellite resonance at 857.7 eV, consistent with the data, whereas the model of Ref. A [8] predicts this feature around 857 eV. Moreover, our model is compatible with the previous optical work, which reported absorption peaks at 1.1, 1.7, 2.2, 3.5, and 4.6 eV [9]. On the contrary, the calculated spectra using the model in Ref. A [8] cannot reproduce the energies of the last three excitations well.

If one considers the exciton in isolation, without requiring a precise reproduction of the shape of the *dd*-excitations, our model and Ref. A [8] both obtain 1.47 eV consistent with the experimental energy of this mode. This observation suggests that there is some ambiguity in determining the model parameters from the exciton energy alone. By analyzing the different models, we found that this ambiguity arises from an anticorrelation between Hund's interactions and charge transfer energy, which produces a ranges of parameters that obtain the observed exciton energy. We note, however, that the Hund's energy is known to be weakly screened in solids and generally falls in a reasonably narrow range of values around 70-90% of the values calculated for isolated atoms [10]. In fact, the onsite Coulomb interaction parameters for Ni 3d orbitals in Ref. A [8], F^2_{dd} and F^4_{dd}, are 120% of atomic values, which is unphysical. Our parameters are 87% of atomic values, which falls within the normal range for correlated insulators.

Although the ground state and exciton in all these models share similar overall symmetry, their electronic composition varies appreciably. For example, our charge-transfer energy (2.50 eV) is larger than that reported in Ref. A (0.95 eV) [8]. Correspondingly, the exciton in our model, which is a singlet, has 1.26 versus 0.74 holes occupying the Ni and S sites, respectively. Conversely, it has 1.08 versus 0.92 holes occupying the Ni and S sites, respectively, in the model of Ref. A [8]. As noted in the main text, the preferential Ni occupation of the exciton is vital for understanding the interactions underlying the exciton formation.

Supplementary Note 2. Wavefuction analysis

As discussed in the main text, we performed a detailed wavefunction analysis for the AIM model to investigate the nature of the exciton, but results are similar for both the AIM and cluster models.

Figure 1**d**–**f** plots several expectation values for the $NiPS_3$ wavefunction, which reveals a significant portion of doubly occupied d^8 configuration and deviation from the pure $d^9\underline{L}$ states. A full understanding of these effects can be obtained by projecting the real wavefunctions onto those devised by Zhang and Rice to describe the $d^9\underline{L}$ manifold [11]. The Zhang-Rice singlet can be written as

$$|\mathrm{ZRS}\rangle = \frac{1}{2}(|d_{x^2-y^2,\downarrow}L_{x^2-y^2,\uparrow}| - |d_{x^2-y^2,\uparrow}L_{x^2-y^2,\downarrow}| + |d_{z^2,\downarrow}L_{z^2,\uparrow}| - |d_{z^2,\uparrow}L_{z^2,\downarrow}|). \tag{2}$$

The Zhang-Rice triplet states are

$$\begin{aligned} |\mathrm{ZRT}, S_z = 1\rangle &= \frac{1}{\sqrt{2}}(|d_{x^2-y^2,\uparrow}L_{z^2,\uparrow}| - |d_{z^2,\uparrow}L_{x^2-y^2,\uparrow}|) \\ |\mathrm{ZRT}, S_z = 0\rangle &= \frac{1}{2}(|d_{x^2-y^2,\uparrow}L_{z^2,\downarrow}| + |d_{x^2-y^2,\downarrow}L_{z^2,\uparrow}| - |d_{z^2,\uparrow}L_{x^2-y^2,\downarrow}| - |d_{z^2,\downarrow}L_{x^2-y^2,\uparrow}|) \\ |\mathrm{ZRT}, S_z = -1\rangle &= \frac{1}{\sqrt{2}}(|d_{x^2-y^2,\downarrow}L_{z^2,\downarrow}| - |d_{z^2,\downarrow}L_{x^2-y^2,\downarrow}|). \end{aligned} \tag{3}$$

Since d^8 states have a significant amount of weight in the wavefunction, these states must also be considered. We

therefore define

$$
\begin{aligned}
|^1A_1, d\rangle &= \frac{1}{\sqrt{2}}(|d_{x^2-y^2,\uparrow}d_{x^2-y^2,\downarrow}| + |d_{z^2,\uparrow}d_{z^2,\downarrow}|) \\
|^3A_2, d, S_z = 1\rangle &= |d_{x^2-y^2,\uparrow}d_{z^2,\uparrow}| \\
|^3A_2, d, S_z = 0\rangle &= \frac{1}{\sqrt{2}}(|d_{x^2-y^2,\downarrow}d_{z^2,\uparrow}| + |d_{x^2-y^2,\uparrow}d_{z^2,\downarrow}|) \\
|^3A_2, d, S_z = -1\rangle &= |d_{x^2-y^2,\downarrow}d_{z^2,\downarrow}|.
\end{aligned} \tag{4}
$$

Similar states exist with two ligand holes as

$$
\begin{aligned}
|^1A_1, L\rangle &= \frac{1}{\sqrt{2}}(|L_{x^2-y^2,\uparrow}L_{x^2-y^2,\downarrow}| + |L_{z^2,\uparrow}L_{z^2,\downarrow}|) \\
|^3A_2, L, S_z = 1\rangle &= |L_{x^2-y^2,\uparrow}L_{z^2,\uparrow}| \\
|^3A_2, L, S_z = 0\rangle &= \frac{1}{\sqrt{2}}(|L_{x^2-y^2,\downarrow}L_{z^2,\uparrow}| + |L_{x^2-y^2,\uparrow}L_{z^2,\downarrow}|) \\
|^3A_2, L, S_z = -1\rangle &= |L_{x^2-y^2,\downarrow}L_{z^2,\downarrow}|.
\end{aligned} \tag{5}
$$

Using this basis, the ground state triplet can be described in terms of the d^8 triplet state mixed with a Zhang-Rice triplet, with only a small contribution from other states (which we denote $|\ldots\rangle$). The wavefunctions are

$$
\begin{aligned}
|\mathrm{GS}, S_z = -1\rangle &= 0.757\ |^3A_2, d, S_z = -1\rangle + 0.163\ |^3A_2, L, S_z = -1\rangle + 0.625\ |\mathrm{ZRT}, S_z = -1\rangle + 0.099\ |\ldots\rangle \\
|\mathrm{GS}, S_z = 0\rangle &= 0.757\ |^3A_2, d, S_z = 0\rangle + 0.163\ |^3A_2, L, S_z = 0\rangle + 0.625\ |\mathrm{ZRT}, S_z = 0\rangle + 0.099\ |\ldots\rangle \\
|\mathrm{GS}, S_z = 1\rangle &= 0.757\ |^3A_2, d, S_z = 1\rangle + 0.163\ |^3A_2, L, S_z = 1\rangle + 0.625\ |\mathrm{ZRT}, S_z = 1\rangle + 0.099\ |\ldots\rangle.
\end{aligned} \tag{6}
$$

The wavefunction of the exciton can be expressed as

$$
|\mathrm{E}\rangle = 0.430\ |^1A_1, d\rangle + 0.209\ |^1A_1, L\rangle + 0.766\ |\mathrm{ZRS}\rangle + 0.430\ |\ldots\rangle, \tag{7}
$$

which demonstrates that the exciton is primarily composed of the singlet components of the d^8, $d^9\underline{L}$, and $d^{10}\underline{L}^2$ states. We also see a noticeable increase in the ligand character compared to the ground state. A further component arises from mixing with a large number of other states.

This wavefunction analysis can further be used to understand the interactions underlying the exciton energy. While the Zhang-Rice singlet and triplet are only split by quite weak exchange interactions, the exciton state has an appreciable fraction of doubly occupied holes, which involve much stronger Hund's exchange. To more accurately quantify the contribution from Hund's interaction J_H, we compute the expectation value of the operator $\hat{J}_H$ for the exciton wavefunction, i.e., $\langle E|\hat{J}_H|E\rangle$. The calculated expectation value is 1.60 eV, which is indeed the leading factor to set the exciton energy scale and compensated slightly by the charge-transfer process to give the exciton energy of 1.47 eV.

Supplementary Note 3. Detailed fitting procedures for the RIXS spectra

As noted in Methods Section, we use Voigt functions to fit the excitons, and use a damped harmonic oscillator (DHO) convoluted with a Gaussian resolution function to fit the magnon as well as the double-magnon peaks. In this section, we provide further details on our fitting procedures.

A. Energy zero determination

To ensure a reliable measurement of the subtle exciton dispersion we observe here, we require an accurate calibration of the energy zero, which is often done by fitting the elastic line. However, in the current case of $NiPS_3$ we only observe quite weak elastic scattering and it is only possible to precisely fit the elastic line in a subset of the spectra. We

consequently use the magnon peak position to perform the fine alignment of the spectra. The expected magnon peak energy is calculated using spin wave theory [12] based on the weighted sum of the magnon branches predicted in the Hamiltonian obtained in Ref. [13]. In this process, structural domain averaging introduces an error of ~ 1 meV and the errors of the neutron results themselves are ~ 2 meV [13, 14]. For the spectra at $|H(K)| \leq 0.1$, because the magnon and elastic peaks are too close to each other, to make the fit converge, we have to manually fix the energy zero to the value that gives the lowest χ^2. In this case, we assign an estimated error bar of 5 meV for the energy zero because the fits would severely deviate from the measured lineshapes if we shifted the energy zero by ± 5 meV or more. The error bars for the fitted double-magnon and exciton peak positions shown in Fig. 2 include not only their own fitting error (0.5–1 meV for the exciton and ~ 3 meV for the double-magnon) but also the fitting error of the energy zero (~ 1 meV) as well as the uncertainty of the calculated magnon energies (model differences and the twinning effect as explained above).

B. Best fits to the low temperature momentum-dependent RIXS spectra

We present the best fits to the linecuts of the RIXS spectra measured at various in-plane momentum transfer at $T = 40$ K in Supplementary Figs. 3–5. Particularly for the low-energy region, we display the three components used in the fits to clarify the identify of different spectral features.

C. Cross-checks of the exciton and double-magnon dispersions

To verify the fitted exciton dispersion, we performed three tests. The first one is to check the consistency with the reported zone-center exciton energy from optical measurements. The exciton energy from previous photoluminescence and optical absorption spectra studies is 1.475 eV with uncertainty below 1 meV [8], which is in line with our fitting results. Even after taking into account the uncertainty from the energy calibration of our spectrometer (~ 2.5 meV at this energy), this zone-center exciton energy taken from optical measurements still has smaller error bar than our fitting results in this region and can help to see an energy difference between the zone-center exciton and excitons at higher $\boldsymbol{Q}$ in Fig. 2. Since the fitted exciton energy heavily depends on the fitting quality of the low-energy region, we next inspect the fitted elastic and magnon peak intensities. As shown in Supplementary Fig. 6, the fitted magnon intensity matches calculated values quite well. The elastic peak becomes stronger near the zone center as expected for specular reflection. The last check we performed is to test the null hypothesis in which we presume that the exciton is, in fact, non-dispersive, and that the apparent dispersion comes from calibration errors. We find that this indeed leads to an unphysical result in which the elastic line intensity drops at the specular $(0, 0)$ position, when it would be expected to increase or stay the same (Supplementary Fig. 6).

Similarly, we also did the null hypothesis test for the double-magnons. Even though the double-magnon peak is broader than the exciton peak and therefore has larger fitting error for the peak positions, we can still exclude the null hypothesis after carefully inspecting the fitted curves. This is most evident in the spectra near the Brillouin zone center, where the fits assuming non-dispersive double-magnons clearly deviate from the best fits and fail to describe the experimental data (Supplementary Fig. 7).

D. Magnon and double-magnon fits with Voigt functions

Although the DHO model are generally used to fit low-energy magnetic excitations in RIXS spectra, to avoid uncertainties inherent in the model we used, we also investigate the Voigt model to fit the magnons and double-magnons. The fitting result turns out to be quite similar to the original DHO fits (Supplementary Fig. 8). Since the magnon peak is quite narrow, both DHO and Voigt models give nearly identical fitting results. Therefore, the change to the energy zero calibration is minimal, so as to the fitted exciton peak positions. For the double-magnon peak, Voigt function gives similar fitting quality but consistently lower peak positions (by ~ 2 meV) as a consequence of increased peak width. However, the existence of the double-magnon dispersion and its resemblance to the exciton dispersion are still valid in the Voigt model fits.

Despite that the low-temperature data set is unable to distinguish the two models, the DHO model is more appropriate to fit the high-temperature data set, where the anti-stoke peaks on the negative energy loss side become more apparent (Supplementary Fig. 9). For consistency, we therefore adopt the DHO model throughout the manuscript to fit the magnon and double-magnon peaks.

E. Fits to the high-temperature spectra

Fitting the high-temperature spectra at 190 K is more difficult since the magnon peak is softened and cannot be used for energy zero determination. Thanks to the enhanced elastic peak, we are able to fit the spectra at several large $\boldsymbol{Q}$ positions (e.g., $H = 0.69$ r.l.u.) where the elastic, magnon, and double-magnon peaks are well separated. The magnon peak is not only softened, but also broadened compared to the low temperature data. We then fix its damping factor z_Q to an average value of 16 meV. Supplementary Fig. 9 is the best fits we obtained for the low-energy region, although the error bars for the energy zero determination could be as large as ~ 5 meV. Then we fit the exciton peak as shown in Supplementary Fig. 10, which has a clear softening and broadening. However, the existence of a dispersive mode is ambiguous here due to the large error bars on the fitted peak positions.

F. Fits to the incident energy dependent spectra

To quantify the resonant behaviors of excitons and double-magnons, we also fit the incident energy dependence of the RIXS spectra to extract their spectral weights (integrated intensities). As expected for a Raman-like process in RIXS the peak energies were seen to be independent of incident energy, so this was used as an additional fitting constraint. In Supplementary Fig. 11, we can see that the magnon peak intensity has only one maximum near the main resonance peak around 853 eV. On the contrary, both the double-magnon peak (Supplementary Fig. 12) and the exciton peak (Supplementary Fig. 13) have two maxima, one near the main resonance peak and the other around 857.7 eV. Such distinct resonance behaviors have been summarized in Fig. 4**a**.

Supplementary Note 4. Double-magnon and exciton propagation

This section provides further analysis of the propagation of the exciton and double-magnon excitations illustrated schematically in Fig. 5 of the main text. For clarity, we will refer to double-magnon propagation via *exchange* and exciton propagation via *hopping*.

A. Double-magnon propagation

$NiPS_3$ exhibits antiferromagnetic order on a honeycomb lattice with both easy-plane and easy-axis anisotropy in addition to first (J_1), second (J_2), and third (J_3) nearest-neighbor isotropic exchange interactions, with the latter being the largest interaction [13, 14]. Despite these complexities, we can obtain a significant amount of insight by considering a simplified picture, which nonetheless captures the essentials of the interactions at play. Propagation within $NiPS_3$'s zig-zag antiferromagnetic ground state can be conceptualized as propagation along either a ferro- or antiferro-magnetic chain direction. We simplify matters further by considering only nearest-neighbor exchange processes in the discussion that follows. (The same procedure applies to longer range exchange processes, as outlined below, so the effects of these terms can also be deduced easily).

For a ferromagnetic chain, the double-magnon propagation occurs via the exchange of $|1,1\rangle$ and $|1,-1\rangle$, where the states are labeled as $|S, m_S\rangle$ following Fig. 5 of the main text. The amplitude for this process can be calculated using second-order perturbation theory in the spin flip processes, with the intermediate state being nearest-neighbor single magnon states with an energy cost proportional to the sum of the single-ion anisotropy $\propto D$ and the spin exchange $\propto J_1$. Altogether, we obtain that such a double-magnon can propagate along the ferromagnetic direction at the scale of the order of $\propto J_1^2/(2D + nJ_1) \sim J_1$, where n is the number of broken magnetic bonds in the intermediate state of

perturbation theory. Although such a calculation is in principle only valid in the Ising-like limit, Ref. [15] has shown that it can be extended also to the isotropic case.

The other propagation direction in the $NiPS_3$ plane is the antiferromagnetic direction (see Fig. 5 of the main text). For this case, we need to invoke a fourth order process in the spin flip terms with three intermediate states, with the highest energy being equivalent to the cost of having two 'extra' double-magnons in a line. This multiplicity is due to the fact that the double-magnon propagation is a composition of two processes along the antiferromagnetic direction, namely 1) the exchange of the $|1,1\rangle$ and $|1,-1\rangle$ states (illustrated in row 2 and 3 of Fig. 5**b** of the main text), and 2) propagation of a double-magnon via an intermediate state with two 'extra' double-magnons on the nearest-neighbor sites. A similar case for the magnon propagation in the antiferromagnet was discussed in Ref. [16]. Due to spin exchanges entering both the denominator and numerator of the perturbative formulae, such a process again leads to an effective propagation at the scale of the spin exchange J_1.

These estimations for the exchange process have implicitly assumed that the double-magnon is a bound state, which is formally justified only in the limit of large Ising anisotropy. For $NiPS_3$, the double-magnon probably has high decay rates into two nearest-neighbor single-magnon states. Fortunately, the latter should remain bound (due to attractive interactions between magnons on nearest neighbor sites), so our analysis should remain a good order-of-magnitude estimate.

We note that the leading J_3 exchange process connects only antiferromagnetically aligned Ni spins, which would suppress any difference between propagation along different directions in the lattice. This is in addition to the fact that $NiPS_3$ is structurally and magnetically twinned, meaning that the ferro- and antiferro-magnetic directions in the lattice are not empirically distinguishable (see the methods section). Although technically very challenging, the development of ultrahigh energy resolution RIXS under strain could be implemented to study specific magnetic monodomains to more directly test whether the double-magnon is or is not a bound state.

B. Exciton hopping

Before discussing the amplitude of the exciton hopping, we first consider possible spin exchanges for the $|1,0\rangle_{\mathbf{i}}$ state with $|1,\pm 1\rangle_{\mathbf{j}}$ between sites $\mathbf{i}$ and $\mathbf{j}$, following the discussion in Ref. [17]. In this case, the relevant terms in the Hamiltonian are

$$\begin{aligned} h^{\text{spin-flip}} &= \frac{J_{\mathbf{i},\mathbf{j}}}{2}\left(S_{\mathbf{i}}^{+}S_{\mathbf{j}}^{-} + \text{h.c.}\right) \\ &= J_{\mathbf{i},\mathbf{j}}\big(|1,1\rangle_{\mathbf{j}}|1,0\rangle_{\mathbf{i}}\ {}_{\mathbf{j}}\langle 1,0|_{\mathbf{i}}\langle 1,1| + |1,-1\rangle_{\mathbf{i}}|1,0\rangle_{\mathbf{j}}\ {}_{\mathbf{i}}\langle 1,0|_{\mathbf{j}}\langle 1,-1| + \text{h.c.}\big), \end{aligned} \tag{8}$$

where $J_{\mathbf{i},\mathbf{j}}$ is the exchange coupling between sites $\mathbf{i}$ and $\mathbf{j}$ and $S_{\mathbf{i}}^{\pm}$ are the raising and lowering spin operators on site $\mathbf{i}$.

We now turn to the exciton hopping process. Since $NiPS_3$ is a magnetically ordered insulator, an exciton at site $\mathbf{i}$, denoted by $|0,0\rangle_{\mathbf{i}}$, can only hop via an interchange process with the $S=1$ states at a neighboring site $\mathbf{j}$ (denoted here as $|1,\pm 1\rangle_{\mathbf{j}}$). The relevant terms in the Hamiltonian for exciton hopping are similar to Eq. (8), i.e.:

$$h^{\text{hop}} = J_{\mathbf{i},\mathbf{j}}\big(|1,1\rangle_{\mathbf{i}}|0,0\rangle_{\mathbf{j}}\ {}_{\mathbf{i}}\langle 0,0|_{\mathbf{j}}\langle 1,1| + |1,-1\rangle_{\mathbf{i}}|0,0\rangle_{\mathbf{j}}\ {}_{\mathbf{j}}\langle 1,-1|_{\mathbf{i}}\langle 0,0| + \text{h.c.}\big), \tag{9}$$

with the prefactors in Eqs. (8) and (9) being identical. The underlying reason for this key observation is that the dominant processes involved are (super)exchange processes that take place on the ligand orbitals rather than the nickel orbitals [17]. As a result, individual $s=1/2$ spin flips cannot happen on the nickel atoms, leading to vanishing amplitudes for terms that would differentiate between the $|0,0\rangle$ singlet hopping and the $|1,0\rangle$ triplet exchange.

C. Exciton propagation

We start by discussing exciton propagation along the ferromagnetic direction in the zigzag antiferromagnetic ground state. In fact, once the exciton hopping is known [see Eq. (9) above], this propagation is the easiest to understand: it merely amounts to the free hopping of the exciton in the ferromagnetic background with a hopping amplitude equal to the spin exchange, similar to the double-magnon case.

A more complex situation is encountered for the antiferromagnetic direction. Here exciton propagation is also due to the hopping process described by Eq. (9) but obtaining a coherent propagation is slightly intricate—as shown in Fig. 5**a**

of the main text. This whole process can essentially be divided into two steps. First, the exciton interchanges twice with the spin background, just as in the ferromagnetic background (second and third row of Fig. 5**a** of the main text). This leads to the creation of an intermediate state with two 'extra' double-magnons situated next to each other. Second, the double-magnons can be annihilated by two spin-exchange processes, in a similar manner as described in Supplementary Note 4 A above. In total, it requires four spin exchanges for the exciton to freely move to the next-nearest-neighbor site, just as the double-magnon does. Such exciton propagation is also at the energy scale proportional to the spin exchange.

Altogether, we observe strong similarities between the way the exciton and the double-magnon move through the spin $S = 1$ zigzag antiferromagnetic honeycomb lattice—in particular, both motions require the same number of spin exchanges and the energy scales are in both cases the same and proportional to the spin exchanges. As discussed in the following section, the dominant exciton hopping is through the third nearest neighbors connected by antiferromagnetically aligned spins, therefore, the dispersions along the in-plane H and K directions would be expected to be overall rather similar, just like the double-magnon case.

Supplementary Note 5. Tight-binding model fit to the exciton dispersion

Tight-binding model approaches have been widely used to model exciton dispersion in molecular solids [18]. Although the way the Hund's exciton we studied here moves is different to the mechanism for Frenkel excitons (spin exchange processes as discussed above instead of dipole–dipole interactions), tight-binding models can still be useful at a phenomenological level to provide a simple, but informative empirical approach to extract the lengthscale of the effective interactions governing the exciton dispersion.

Using the monoclinic unit-cell notation, we formulated a simplified effective tight-binding model on a two-dimensional honeycomb lattice with isotropic effective "hopping" terms t_n. We obtain three terms in the exciton dispersion (E_{t1}, E_{t2}, and E_{t3}) associated with first, second, and third nearest neighbor effective interactions (t_1, t_2 and t_3), where

$$\begin{aligned} E_{t1} =& t_1 \sqrt[\pm]{4\cos^2(\pi H) + 4\cos(\pi H)\cos(\pi K) + 1} \\ E_{t2} =& 2t_2\{\cos(2\pi H) + \cos[\pi(H+K)] + \cos[\pi(H-K)]\} \\ E_{t3} =& t_3 \sqrt[\pm]{4\cos^2(2\pi H) + 4\cos(2\pi H)\cos(2\pi K) + 1}. \end{aligned} \tag{10}$$

By co-fitting the measured low-temperature exciton dispersion along both H and K directions, we obtain the results as shown in Supplementary Fig. 14. For E_{t1} and E_{t3}, we selected the sign of the solution based on the empirically observed upward-dispersion trend near the Brillouin zone center. We tested the individual contribution of the three different functional forms and found that the fits with third nearest neighbor effective hopping alone can capture the observed periodicity of the dispersion, indicating that third nearest neighbor interactions play the leading role in the exciton dispersion. As stated before, this phenomenological model is an effective parameterization of a process that fundamentally arises from magnetic exchange and not real hopping. Our observation of leading third nearest neighbor interactions is consistent with the fact the third nearest neighbor spin exchange is dominant in the spin Hamiltonian of $NiPS_3$ [13, 14].

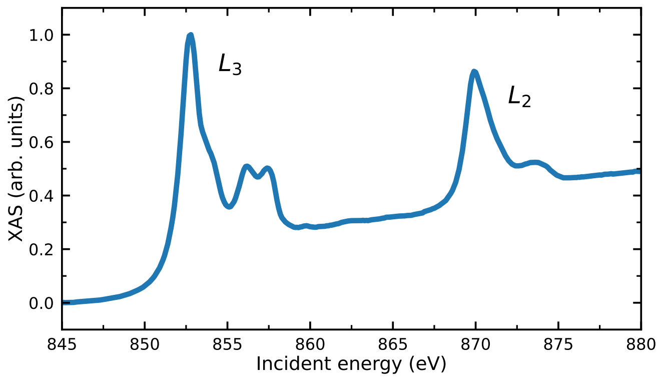

Supplementary Figure 1. **XAS spectra**. The spectra was taken at 40 K using π-polarized x-rays in the total fluorescence yield mode. The sample geometry was the same as the measurement for the RIXS energy map (i.e., $\theta = 22.6°$, $2\Theta = 150°$, $(0KL)$ scattering plane) except that the photo diode detector was placed slightly above the scattering plane.

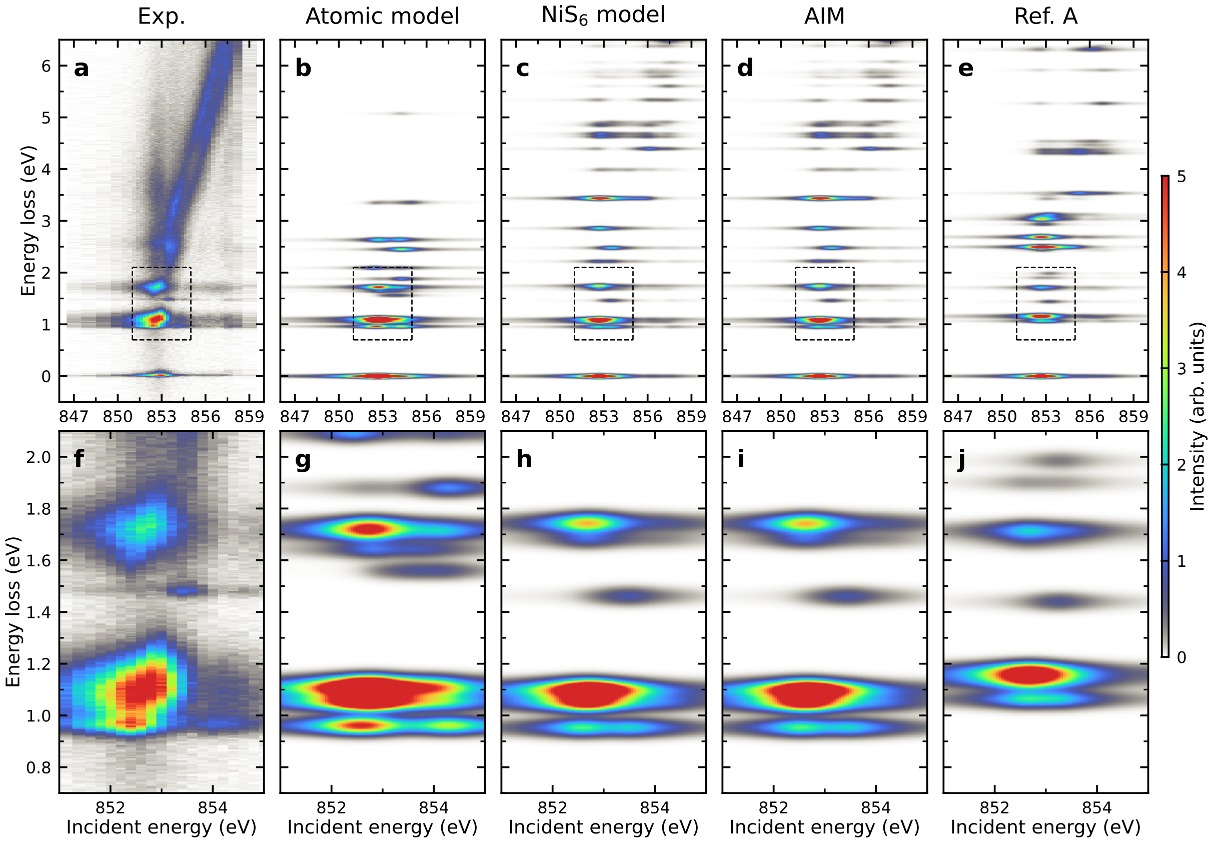

Supplementary Figure 2. **Comparison of RIXS energy maps calculated using different models**. **a**–**e**, The measured and calculated RIXS energy maps. **f**–**j**, Zoom-in of the measured and calculated RIXS maps in the region outlined by the dashed box in panels **a**–**e**. The calculations assume a constant lifetime for the excitations, and omit the continuum of states needed to accurately simulate the fluorescent processes that occur at high energies, so the transitions above 2 eV appear far narrower in the calculations compared to the theory.

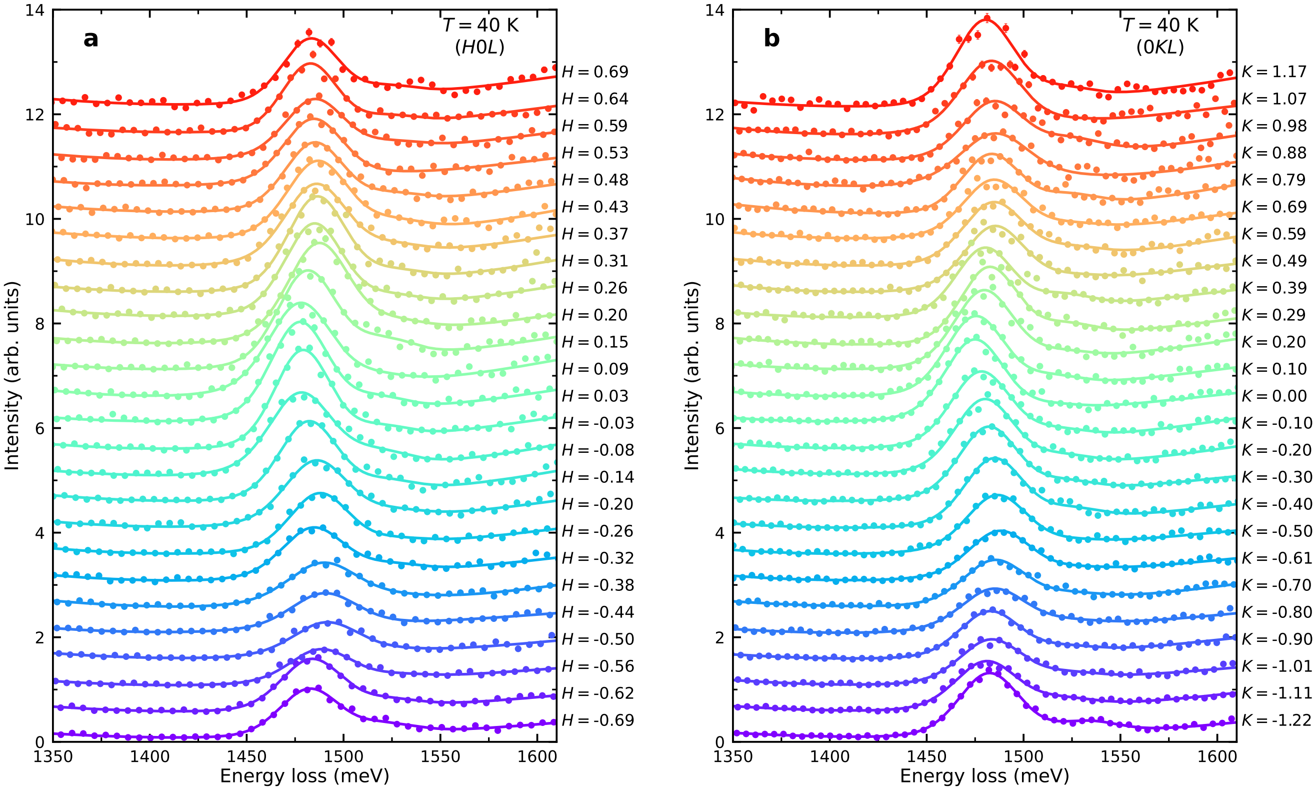

Supplementary Figure 3. **RIXS spectra measured at $T = 40$ K with an energy window chosen to isolate the exciton**. **a**, The exciton peak at various in-plane momentum transfer H measured in the $(H0L)$ plane. **b**, The exciton peak at various in-plane momentum transfer K measured in the $(0KL)$ plane. All the measurements were taken with linear horizontal π polarization of the incident x-rays at the resonant energy of 853.4 eV for excitons. The data is the same as the intensity maps shown in Fig. 2**a** and **b**. The solid lines are fits to the data with details in Methods Section. Data are shifted vertically for clarity. Error bars represent one standard deviation.

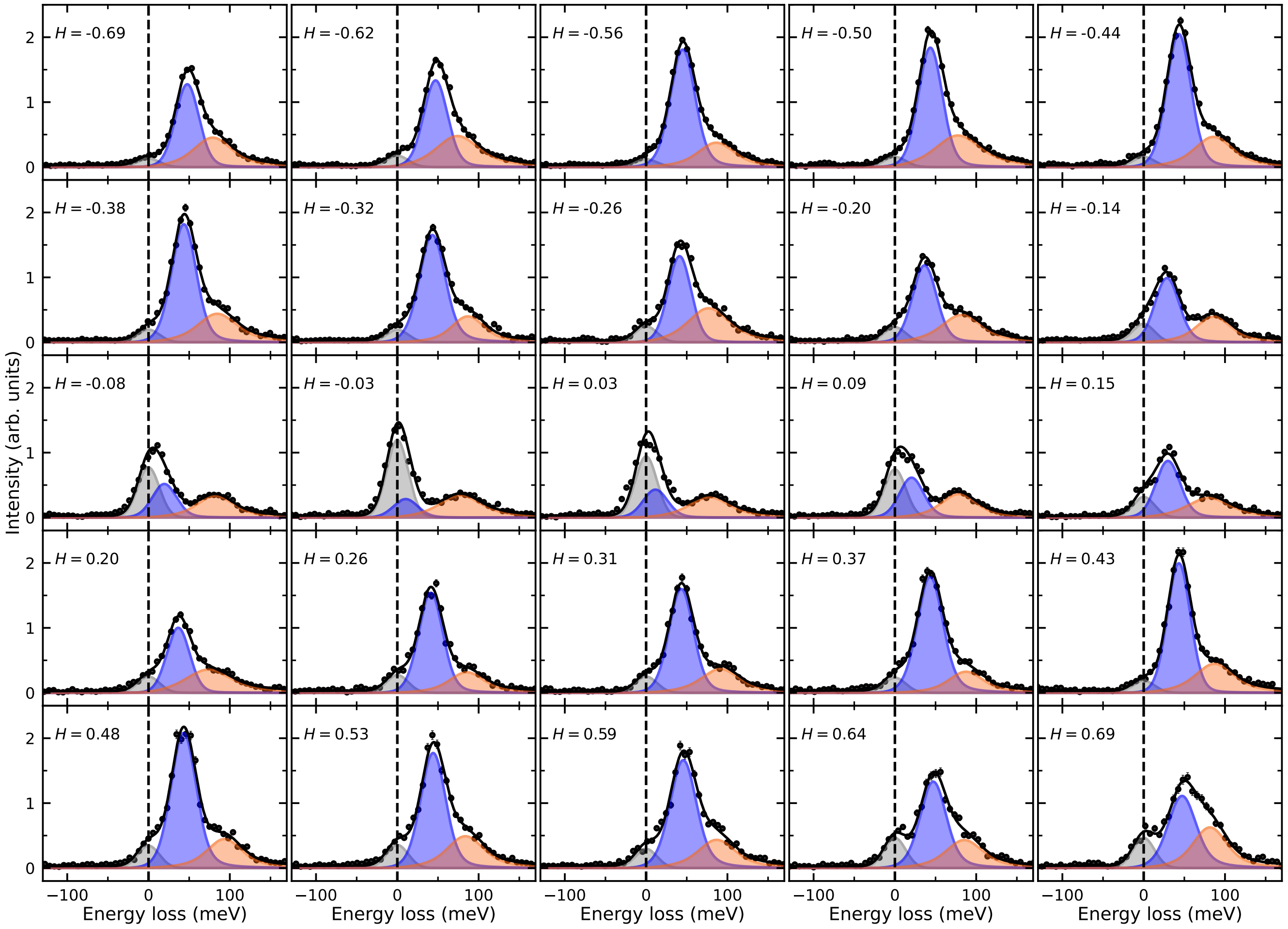

Supplementary Figure 4. **RIXS spectra measured in the ($H0L$) plane at $T = 40$ K with an energy window chosen to isolate the magnon and double-magnon**. Each panel displays the spectra at a specific in-plane momentum transfer H measured in the ($H0L$) plane. The solid lines are best fits to the data with three components, i.e., the elastic line (gray), the magnon peak (blue), and the double-magnon peak (orange). The detailed description of the fitting can be found in the Methods Section and Supplementary Note 3. The vertical dashed line in each panel labels the energy zero. Error bars represent one standard deviation.

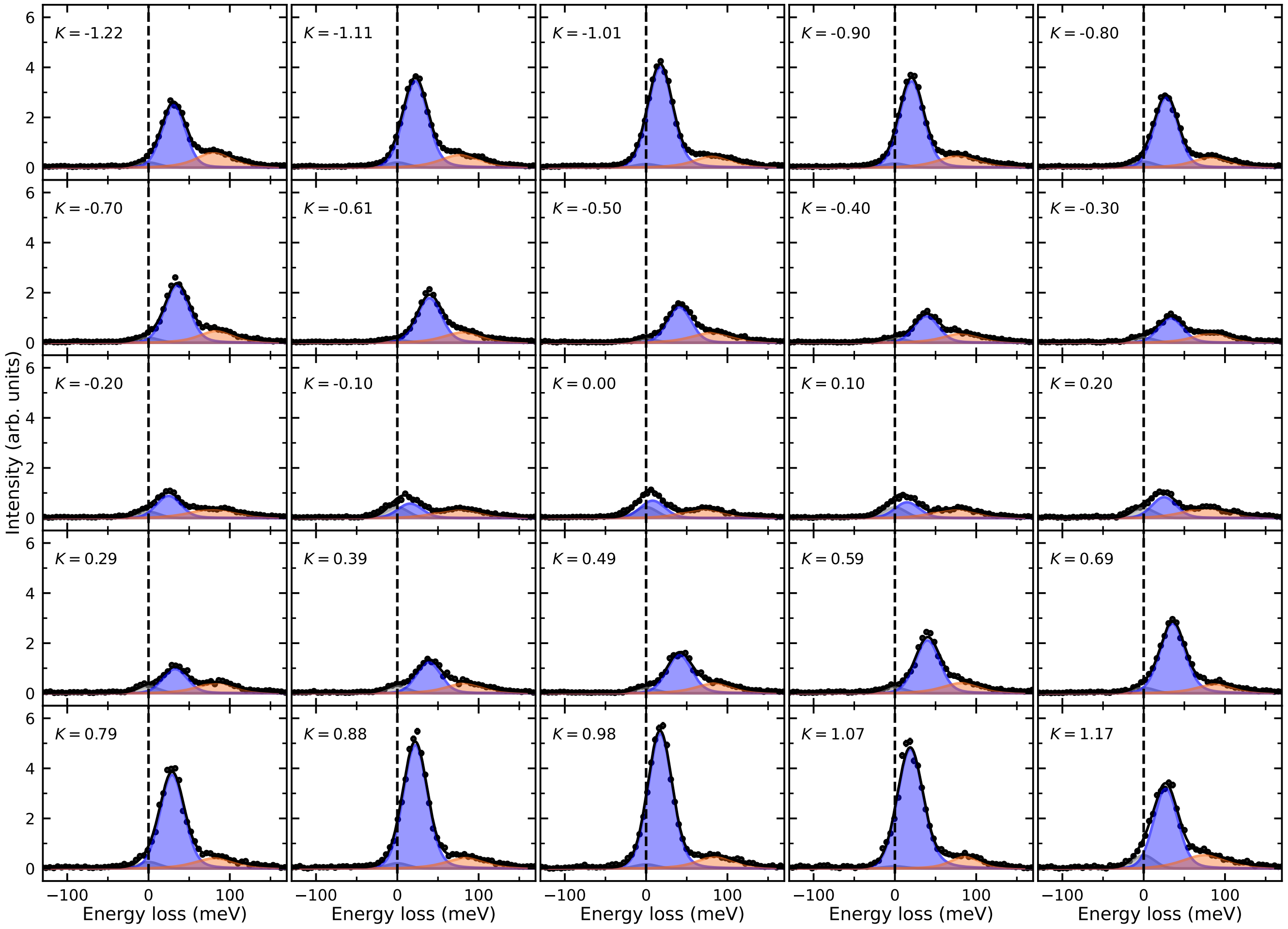

Supplementary Figure 5. **RIXS spectra measured in the $(0KL)$ plane at $T = 40$ K with an energy window chosen to isolate the magnon and double-magnon**. Each panel displays the spectra at a specific in-plane momentum transfer K measured in the $(0KL)$ plane. The solid lines are best fits to the data with three components, i.e., the elastic line (gray), the magnon peak (blue), and the double-magnon peak (orange). The detailed description of the fitting can be found in the Methods Section and Supplementary Note 3. The vertical dashed line in each panel labels the energy zero. Error bars represent one standard deviation.

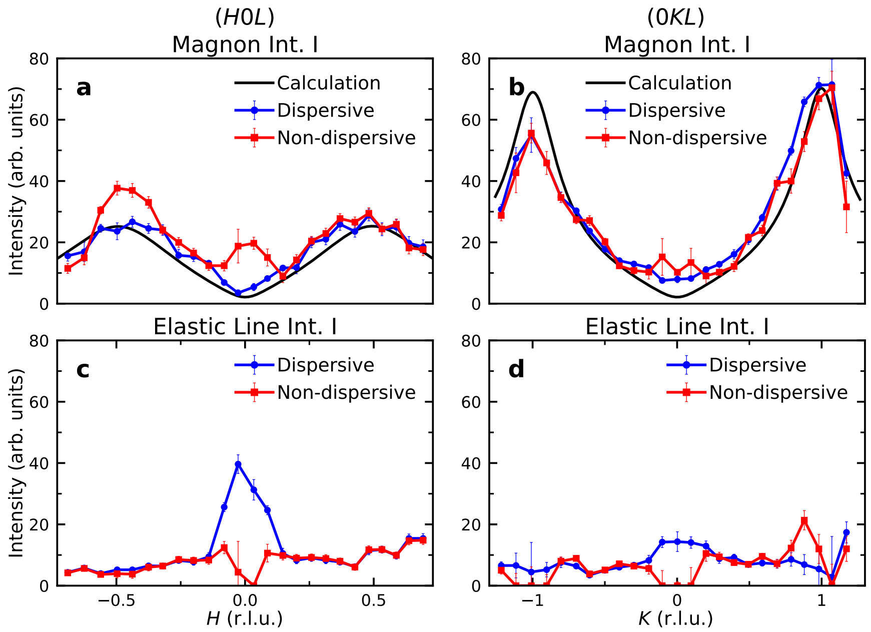

Supplementary Figure 6. **Fitting result comparison between the best fit (blue circle), which gives a dispersive exciton mode, and the fit assuming a null hypothesis of a non-dispersive exciton (red square) coming from a miscalibration of the spectra**. **a**–**b**, Integrated Intensity of the magnon peak measured in $(H0L)$ plane **a** and $(0KL)$ plane **b**, respectively. The black solid lines are the calculated magnon intensities as described in the Methods Section, with the same scaling factor for both panels. **c**–**d**, Integrated Intensity of the elastic peak measured in $(H0L)$ plane **c** and $(0KL)$ plane **d**, respectively. We see that the best fit is in good accord with the calculated magnon intensity, whereas the null hypothesis can be excluded as it implies an unphysical decrease in the elastic line intensity at $(0, 0)$, which is the point at which the elastic line intensity would usually be maximized.

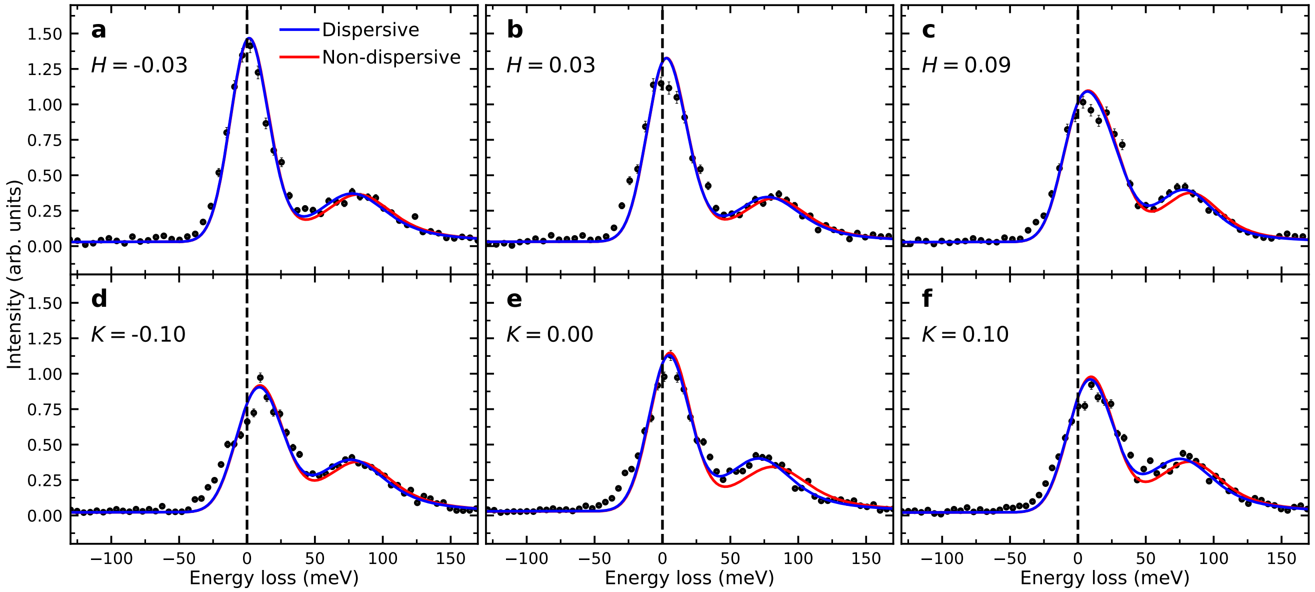

Supplementary Figure 7. **Representative fitting comparisons between the best fits (blue lines), which give a dispersive double-magnon mode, and the fits assuming a null hypothesis of a non-dispersive double-magnon (red lines)**. **a**–**c**, RIXS spectra measured in the $(H0L)$ plane at $T = 40$ K at three representative in-plane momentum transfers H. **d**–**f**, RIXS spectra measured in the $(0KL)$ plane at $T = 40$ K at three representative in-plane momentum transfers K. We can clearly see that red lines assuming non-dispersive double-magnons are worse compared to the best fits (blue lines) which give dispersive double-magnons. The vertical dashed line in each panel labels the energy zero. Error bars represent one standard deviation.

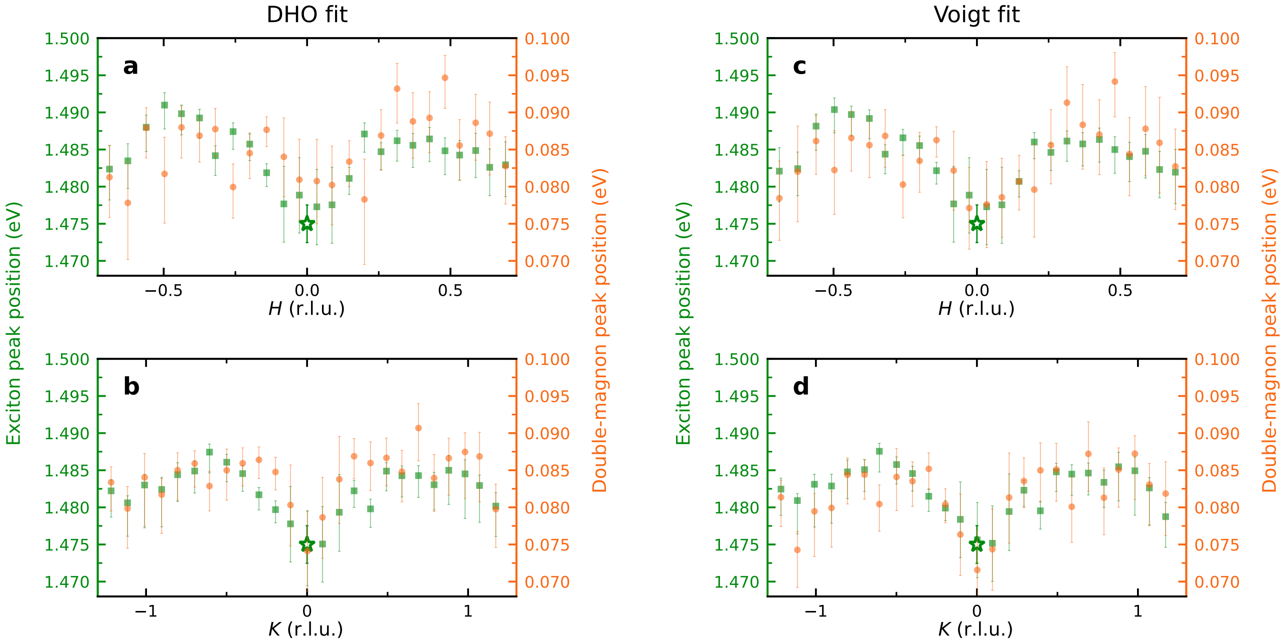

Supplementary Figure 8. **Fit comparison between the DHO and Voigt models used for the magnon and double-magnon peaks**. **a**–**b**, fitted peak positions of the excitons (green squares) and double-magnons (orange circles) using the DHO model for the magnon and double-magnon peaks. The data is the same as the dispersion plots shown in Fig. 2**e** and **f**. **c**–**d**, fitted peak positions of the excitons (green squares) and double-magnons (orange circles) using the Voigt function for the magnon and double-magnon peaks. The fitting results based on the two different functional forms for the magnons and double-magnons are quite similar, validating the robustness of the exciton and double-magnon dispersions.

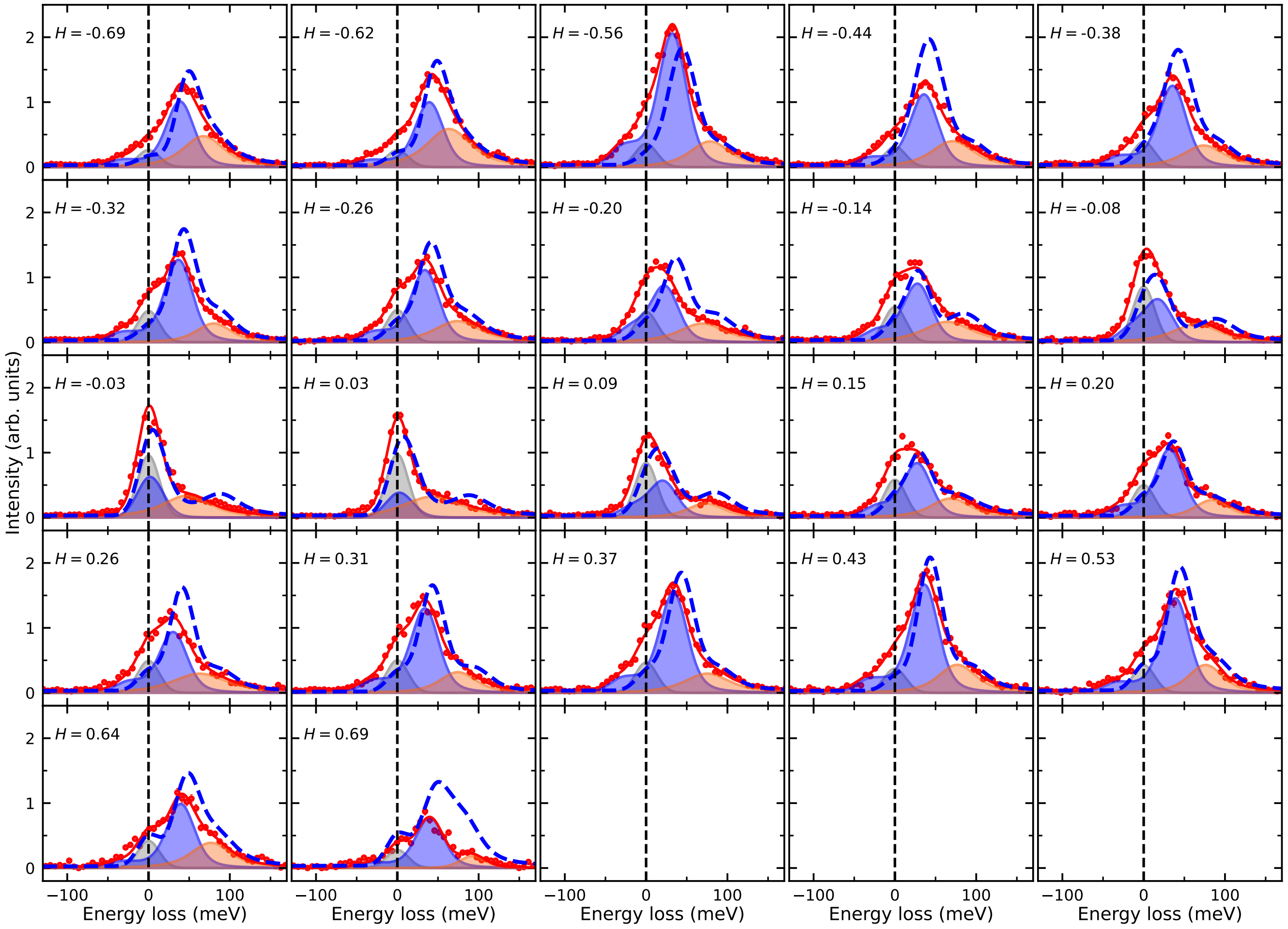

Supplementary Figure 9. **RIXS spectra measured in the $(H0L)$ plane at $T = 190$ K with an energy window chosen to isolate the magnon and double-magnon**. Each panel displays the spectra at a specific in-plane momentum transfer H measured in the $(H0L)$ plane at $T = 190$ K, above its magnetic ordering temperature. We used linear horizontal π polarization of the incident x-rays at the resonant energy of 853.4 eV for excitons. These data are the same as the intensity maps shown in Fig. 3**b** and are provided to show the linecuts directly. The solid red lines are best fits to the data with three components, i.e., the elastic line (gray), the magnon peak (blue), and the double-magnon peak (orange). These are provided to clarify the identity of different spectral features. The blue dashed line shows fits to the 40 K data for comparison. The vertical dashed line in each panel labels the energy zero. Error bars represent one standard deviation.

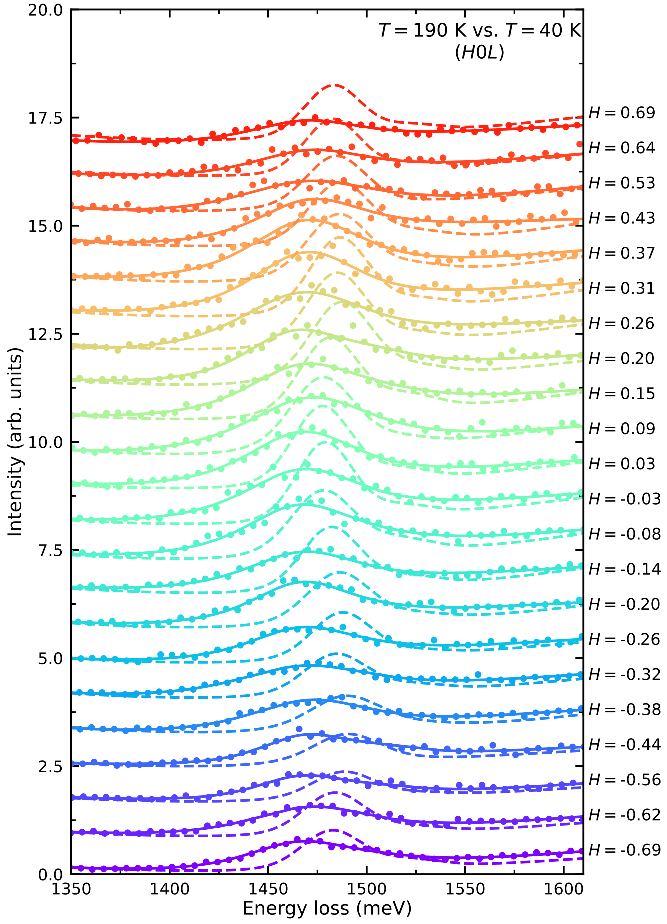

Supplementary Figure 10. **RIXS spectra measured at T = 190 K with an energy window chosen to isolate the exciton**. The measurements were taken at various in-plane momentum transfer H in the $(H0L)$ plane at $T = 190$ K, above its magnetic ordering temperature. We used linear horizontal π polarization of the incident x-rays at the resonant energy of 853.4 eV for excitons. These data are the same as the intensity maps shown in Fig. 3**a** and are provided to show the linecuts directly. The solid lines are fits to the data. The dashed lines show the fitted curves to the corresponding 40 K data for comparison. Data are shifted vertically for clarity. Error bars represent one standard deviation.

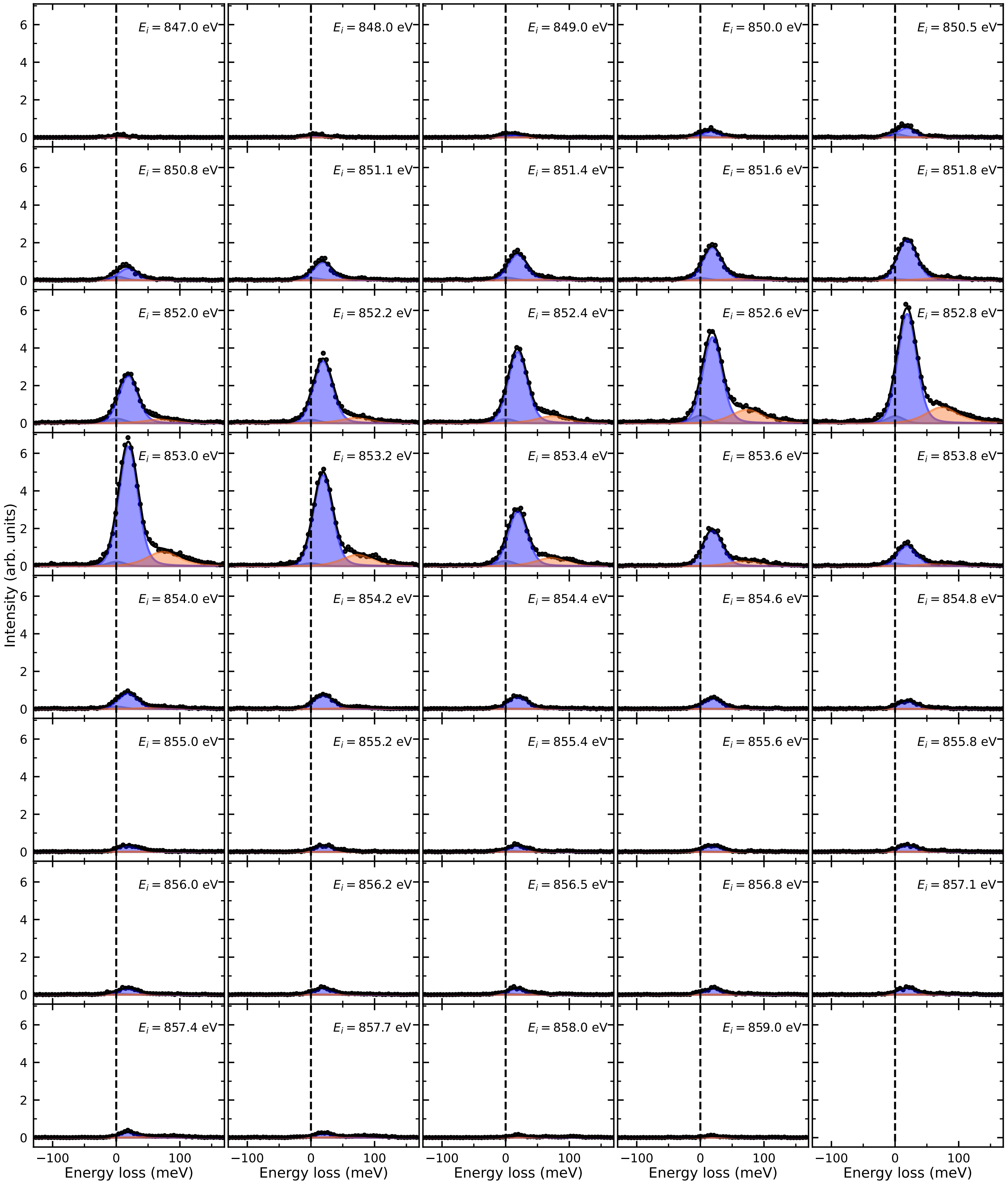

Supplementary Figure 11. **Incident energy dependence of the RIXS spectra with an energy window chosen to highlight the intensity change of the magnon peak**. Each panel displays the spectra with a specific incident photon energy through the Ni L_3 resonance measured in the $(0KL)$ plane at $T = 40$ K with linear horizontal π polarization of the incident x-rays. These data are the same as the intensity maps shown in Fig. 1**a** and are provided to show the linecuts directly. The solid red lines are best fits to the data with three components, i.e., the elastic line (gray), the magnon peak (blue), and the double-magnon peak (orange). The vertical dashed line in each panel labels the energy zero. Error bars represent one standard deviation.

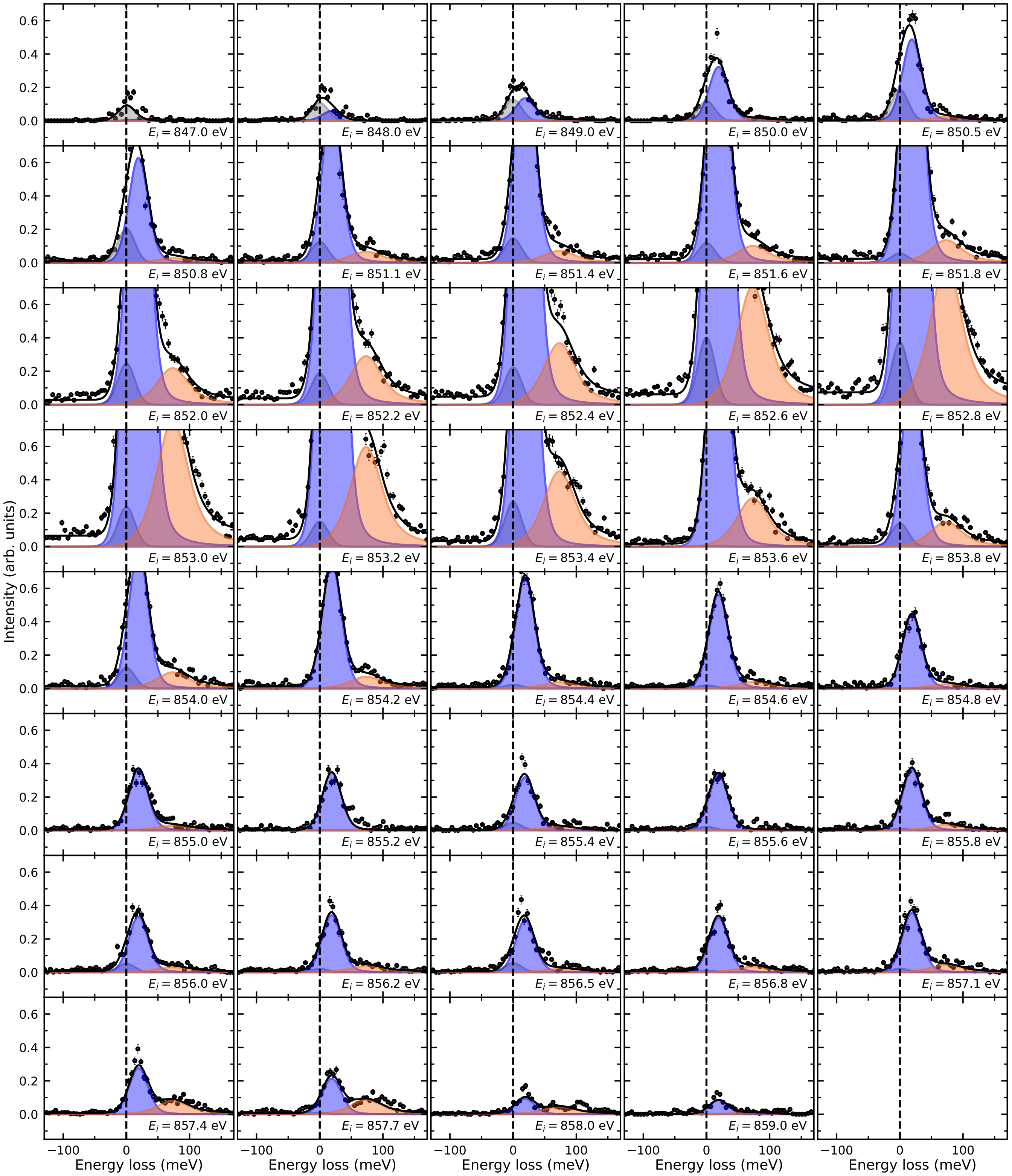

Supplementary Figure 12. **Incident energy dependence of the RIXS spectra with an energy window chosen to highlight the intensity change of the double-magnon peak**. Each panel displays the spectra with a specific incident photon energy through the Ni L_3 resonance measured in the $(0KL)$ plane at $T = 40$ K with linear horizontal π polarization of the incident x-rays. These data are the same as that shown in Supplementary Fig. 11 but zoomed in on the double-magnon peak (orange). Error bars represent one standard deviation.

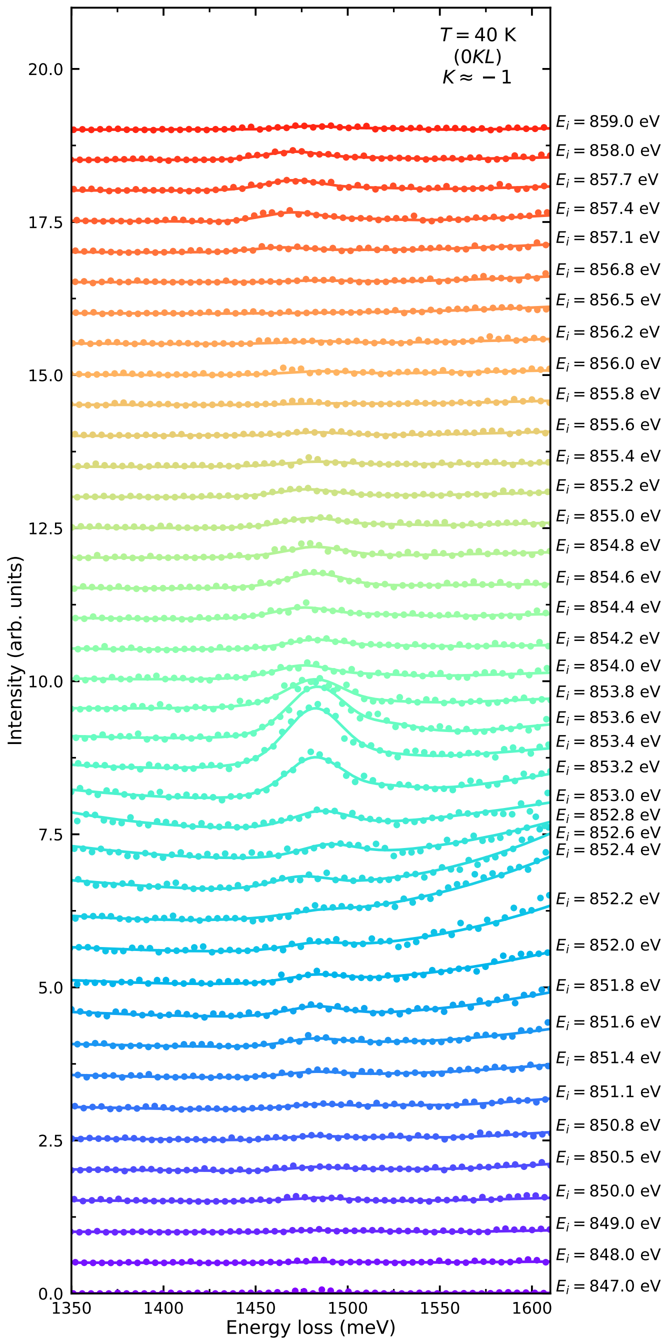

Supplementary Figure 13. **Incident energy dependence of the RIXS spectra with an energy window chosen to highlight the intensity change of the exciton peak**. The measurements were taken at various incident photon energy through the Ni L_3 resonance measured in the $(0KL)$ plane at $T = 40$ K with linear horizontal π polarization of the incident x-rays. These data are the same as the intensity maps shown in Fig. 1**a** and are provided to show the linecuts directly. The solid lines are the fits to the data with details in Methods Section. Data are shifted vertically for clarity. Error bars represent one standard deviation.

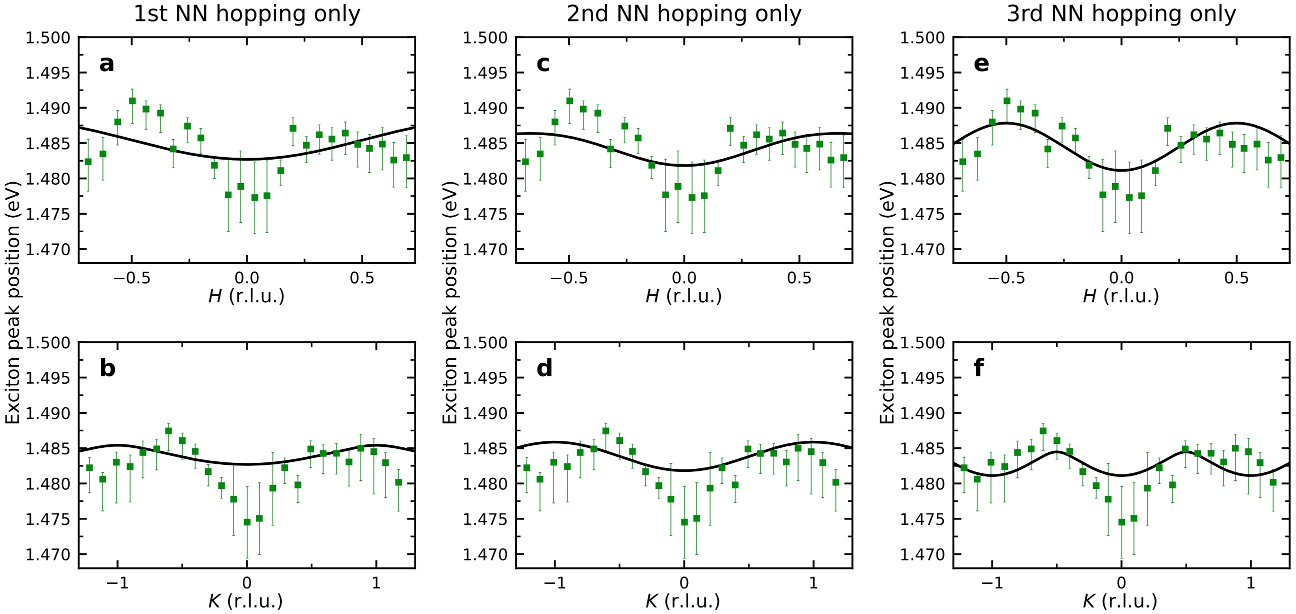

Supplementary Figure 14. **Tight-binding model fits to the exciton dispersion**. Each column displays tight-binding model fits with only first (**a**–**b**), second (**c**–**d**), and third (**e**–**f**) nearest neighbor interactions, respectively. The green squares are the measured exciton dispersion as a function of the H and K in-plane momentum transfer, respectively. The black lines are fitted curves. The best fitted values are $t_1 = 1.4(5)$ meV, $t_2 = -0.5(1)$ meV, and $t_3 = 1.7(3)$ meV, respectively. Error bars represent one standard deviation.

[1] Afanasiev, D. *et al.* Controlling the anisotropy of a van der Waals antiferromagnet with light. *Science Advances* **7**, eabf3096 (2021).
[2] Chandrasekharan, N. & Vasudevan, S. Magnetism and exchange in the layered antiferromagnet $NiPS_3$. *Journal of Physics: Condensed Matter* **6**, 4569 (1994).
[3] Wang, Y., Fabbris, G., Dean, M. & Kotliar, G. EDRIXS: An open source toolkit for simulating spectra of resonant inelastic x-ray scattering. *Computer Physics Communications* **243**, 151–165 (2019).
[4] Neudert, R. *et al.* Four-band extended Hubbard Hamiltonian for the one-dimensional cuprate Sr_2CuO_3: Distribution of oxygen holes and its relation to strong intersite Coulomb interaction. *Phys. Rev. B* **62**, 10752–10765 (2000).
[5] Mizuno, Y. *et al.* Electronic states and magnetic properties of edge-sharing Cu-O chains. *Phys. Rev. B* **57**, 5326–5335 (1998).
[6] Eskes, H., Tjeng, L. H. & Sawatzky, G. A. Cluster-model calculation of the electronic structure of CuO: A model material for the high-T_c superconductors. *Physical Review B* **41**, 288–299 (1990).
[7] Haverkort, M. W., Zwierzycki, M. & Andersen, O. K. Multiplet ligand-field theory using Wannier orbitals. *Physical Review B* **85**, 165113 (2012).
[8] Kang, S. *et al.* Coherent many-body exciton in van der Waals antiferromagnet $NiPS_3$. *Nature* **583**, 785–789 (2020).
[9] Kim, S. Y. *et al.* Charge-Spin Correlation in van der Waals Antiferromagnet $NiPS_3$. *Physical Review Letters* **120** (2018).
[10] Khomskii, D. *Transition metal compounds* (Cambridge University Press, 2014).
[11] Zhang, F. C. & Rice, T. M. Effective Hamiltonian for the superconducting Cu oxides. *Physical Review B* **37**, 3759–3761 (1988).
[12] Toth, S. & Lake, B. Linear spin wave theory for single-Q incommensurate magnetic structures. *Journal of Physics Condensed Matter* **27** (2015).
[13] Scheie, A. *et al.* Spin wave hamiltonian and anomalous scattering in $NiPS_3$. *Phys. Rev. B* **108**, 104402 (2023).
[14] Wildes, A. R. *et al.* Magnetic dynamics of $NiPS_3$. *Physical Review B* **106**, 174422 (2022).
[15] Zheng, W., Oitmaa, J. & Hamer, C. J. Series studies of the spin-$\frac{1}{2}$ Heisenberg antiferromagnet at $T = 0$: Magnon dispersion and structure factors. *Phys. Rev. B* **71**, 184440 (2005).
[16] Verresen, R., Pollmann, F. & Moessner, R. Quantum dynamics of the square-lattice Heisenberg model. *Phys. Rev. B* **98**, 155102 (2018).
[17] Takubo, K. *et al.* Unusual superexchange pathways in an NiS_2 triangular lattice with negative charge-transfer energy. *Physical Review Letters* **99**, 037203 (2007).
[18] Cudazzo, P., Sottile, F., Rubio, A. & Gatti, M. Exciton dispersion in molecular solids. *Journal of Physics: Condensed Matter* **27**, 113204 (2015).
